# The *fos* homolog *kayak* is required for adult eye formation and function in *Drosophila*

**DOI:** 10.3389/fnins.2025.1703753

**Published:** 2026-01-14

**Authors:** Manuel Zúniga-García, Juan Rafael Riesgo-Escovar

**Affiliations:** 1 Laboratorio D-04, Departamento de Neurobiología del Desarrollo y Neurofisiología, Instituto de Neurobiología, Universidad Nacional Autónoma de México, Querétaro, Mexico; 2 Posgrado en Ciencias Biológicas, Unidad de Posgrado, Ciudad Universitaria, Universidad Nacional Autónoma de México, Coyoacán, Mexico

**Keywords:** behavior, *Drosophila melanogaster*, eye, *fos*, *kayak*, sensory morphology

## Abstract

This study characterizes the requirements of the *kayak* (*kay*) gene in *Drosophila melanogaster* adult eye biology by examining mutant phenotypes in photoreceptor development, external eye morphology, corneal and bristle ultrastructure, and visually guided behaviors, such as phototaxis, in *kay* strong loss-of-function homozygous mutant clones. Despite previous studies on *kay*, there is a dearth of phenotypic characterization of the morphological and behavioral consequences of *kay* loss-of-function alleles in the adult eye. We find that *kay* is expressed in developing ommatidia in eye discs. The *kay* mutant ommatidia are misaligned, lack photoreceptors, have malformed corneal surfaces, and have misshaped, misplaced, and fewer mechanosensory bristles. Corneal nipples, while present in mutant corneas on the corneal surface, are disorganized and malformed. With an average of 30% of the eye territory mutant, flies have a significantly lower response in a behavioral phototaxis assay. Altogether, *kay* function is required for multiple cell types in the adult retina, and this stands in stark contrast with other jun kinase genes, like the fly homologs of *jun kinase* and *jun*, genes not required for adult eye morphogenesis. This is consistent with Kayak functions that are independent of heterodimerizing with Jun proteins or requiring activation of the jun kinase pathway.

## 1 Introduction

The *fos* genes, an evolutionarily conserved family of genes, have been associated with a myriad of functions. Fos proteins contain two extremely conserved domains, a basic region for DNA binding to the associated trans regulatory element (TGACTCAG) (Rauscher et al., 1988), and a leucine zipper for protein–protein interaction (Kang et al., 2019). They are generally thought to act as dimers, many times partnering with transcription factors with similar leucine zipper and basic domains of the Jun family (Alfonso-Gonzalez and Riesgo-Escovar, 2018; Zuniga-Garcia and Riesgo-Escovar, 2025).

Originally, a viral form of *c-fos*, *v-fos*, was isolated over 60 years ago as a virally encoded oncogene capable of cellular transformation (Finkel et al., 1966). It was demonstrated afterward that the cellular counterpart (*c-fos*) is present in the genomes of many organisms. The oncogenic form was a mutated version of the cellular gene (Curran et al., 1984; Miller et al., 1984). Most of the published studies to date characterizing *fos* genes have employed mRNA and protein ectopic expression and/or gain-of-function studies (Kovacs, 1998). Far fewer studies have used loss-of-function approaches to address requirements (Johnson et al., 1992). The expression levels reached in different reports vary (due to redundancy, as is the case for vertebrate *fos* genes, or the strength of the expression system, for example), leading to differing results in phenotypic variations and compensatory action (Alfonso-Gonzalez and Riesgo-Escovar, 2018).

In general, *fos* genes code for proteins that act as transcription factors that are induced early and transiently by many different stimuli [i.e., *c-fos* is an example of an immediate early gene (Sheng and Greenberg, 1990)], so much so that they are routinely used to assess activation of cells, especially in the nervous system. Examples include activation of *c-fos* after learning and memory in the hippocampus (Minatohara et al., 2015) or exercise in the same brain region (Rahmi et al., 2024). In addition to this well-known activation, c-Fos can be involved in the cytoplasm as an enzyme in lipid synthesis (Caputto et al., 2014; Caputto and Guido, 2000), or the *Drosophila fos* homolog, *kayak*, cDNA, as a source for piwi-interacting RNAs (Klein et al., 2016). The consequences of *c-fos* expression are generally thought to lead to expression of later-acting genes, but pinpointing which genes and ascribing phenotypes to loss-of-function conditions for *fos* has not been very forthcoming (Zhang et al., 2002; Yasoshima et al., 2006). For this reason, a well-studied and known system where clear consequences for *fos* loss-of-function conditions could be assessed is desirable. *Drosophila* has only one *fos* homolog named *kayak* (*kay*) (Jurgens et al., 1984; Riesgo-Escovar and Hafen, 1997a), and the fly adult compound eye is such a very well-studied system (Baker et al., 2014).

The adult Drosophila eye is a compound eye; that is, it is an organ composed of an array of smaller optical units, the ommatidia, where each one is constituted of a few stereotypical cell types (Bate and Martinez Arias, 1993). Each ommatidium or unit eye consists of a small complement of cells that differentiate sequentially during development. In the center are photoreceptor cells, where six outer photoreceptors (R1–R6) whose rhabdomeres, or light-sensing organelles, surround the rhabdomeres of two internal photoreceptors (R7 and R8). Surrounding these cells are pigment cells—first two primary pigment cells, then six secondary pigment cells in an outer circle—interspersed with either tertiary pigment cells (three, on alternate corners) between secondary pigment cells or mechanosensory bristles (also three, interspersed on alternate corners between secondary pigment cells). On top of the photoreceptors and pigment cells are four cone cells that give rise to the ommatidial cornea (Kumar, 2012). The mechanosensory bristles develop from a precursor cell by two unequal mitotic divisions to give rise to four cells: a neuron, a glial cell, a socket, and a hair cell; the last two form the bristle and then die (Meserve and Duronio, 2017). It has been a model organ in large part due to its near-crystalline array of ommatidia. It allows for even very subtle phenotypes due to mutations or genetic manipulations to be noticed and characterized. Also, a large array of tools has been developed specifically to study its development and function (Weasner and Kumar, 2022).

Both the Jun and Fos fly homologs (*Jra* and *kay*, respectively) have been characterized and shown to partake in the Jun kinase signal transduction pathway (Riesgo-Escovar and Hafen, 1997a,b). They act in concert in many instances in the fly, like during dorsal and thoracic closures. Yet, in stark contrast, both the Jun kinase gene and the Jun homolog gene in flies are not required for eye formation (Riesgo-Escovar et al., 1996; Riesgo-Escovar and Hafen, 1997b). In fact, there are examples during development where *kay* is required, but not *Jra* (Riesgo-Escovar and Hafen, 1997a; Szuts and Bienz, 2000; Dequier et al., 2001). Drosophila adult eye formation and function, as we report here, is another instance where only *kay* is required, but not *Jra*, or *basket*, the last being the jun kinase homolog in flies (Riesgo-Escovar et al., 1996).

Here, we employ strong loss-of-function mutations in the sole gene coding for a Fos-family of transcription factors in the genome of the fruit fly, the gene called *kayak* (*kay*) (Riesgo-Escovar and Hafen, 1997a), to address requirements during adult eye formation. We make use of genetic mosaics, effectively generating organisms where only part of the retinal tissue is homozygous mutant, and the rest of the organism is heterozygous, and thus, of a wild-type phenotype (*kay* mutations are recessive). The *kay* mutations used here are homozygous embryonic lethal. We take advantage of the fact that only part of the eye is homozygous mutant for *kay* since it allows us to compare directly with control tissue in the same organisms, with practically the same genetic background, having developed in the same environment and at the same time. We find that *kay* is expressed during eye formation and is required in multiple instances during eye development.

## 2 Materials and methods

### 2.1 Fly stocks

Fly stocks were cultured on standard unrefined sugar-yeast medium at 25 °C, 40–50% relative humidity, in a 12:12 light: dark cycle. *Drosophila melanogaster* stocks used for generating eye clones were BDSC #43348 and BDSC #5619 (BDSC is Bloomington Drosophila Stock Center) according to Gambis et al. (2011). The mutant *kayak* (*kay*) alleles analyzed were *kay^1^, kay^5^,* and *kay^2^. kay^1^* is a strong loss-of-function, EMS-induced nonsense mutation (Jurgens et al., 1984; Riesgo-Escovar and Hafen, 1997a) (Supplementary Figure 1A).

*kay^5^*, originally described as *l(3)s064007/82* in (Bellotto et al., 2002), is a P-element transposon insertion and also a strong loss-of-function mutation derived from another mutant screen (with a different genetic background and many years apart from the one where *kay^1^* and *kay^2^* were isolated) (Bellotto et al., 2002), thus making it unlikely that a second site linked mutation is responsible for the mutant phenotypes described in this article for *kay^1^*, *kay^2^*, and *kay^5^*. We did an inverse PCR with 5’ P element primers, exactly as described in Huang et al. (2009), and cloned the genomic region next to the insertion. The insertion is in the first intron of the locus, disrupting an enhancer sequence for the Eyeless transcription factor (Zou et al., 2024); (Supplementary Figure 1A).

In addition to these strong loss-of-function mutant alleles, we also used *kay^2^*, BDSC #42217. *kay^2^* is an EMS-induced mutation, as *kay^1^* [from the same mutagenesis (Jurgens et al., 1984)], but its molecular lesion has not been localized. It is a hypomorphic mutation of the locus since some homozygotes can survive to adulthood when reared at 18 °C (Zeitlinger et al., 1997). All three alleles fail to complement each other in embryonic lethality at 25 °C, and a *kay^1^/kay^2^* trans-heterozygote embryo is rescued to adulthood by a *kay* transgene (Riesgo-Escovar and Hafen, 1997a). To control for second-site mutations or accumulation of modifiers, especially given the time elapsed between the mutant alleles isolation, and this study, all three mutant alleles were backcrossed to the same genetic background, a *yellow, white* control strain, by repeatedly recombining them to a *yellow, white*; FRT82 stock, also to be able to generate mutant eye clones. Since *kay* loss-of-function alleles are embryonic lethal, like *kay^1^* and *kay^5^* (*kay^2^* homozygotes are completely lethal when reared at 25 °C), stocks are maintained heterozygous over a “balancer chromosome.” This balancer chromosome was introduced with the same genetic background and has a dominant marker, is homozygous lethal, and prevents recombination with the homologous chromosome carrying the *kay* mutation. Finally, we also used a *kay* protein trap that has a wild-type phenotype, inserted in the fourth intron of the locus (Ozturk-Colak et al., 2024), and listed as BDSC #36175 (Supplementary Figure 1A), to evidence expression of Kayak with the *yellow, white* (*y.w*) stock as control flies. For full genotypes, refer to Supplementary Table 1.

### 2.2 Genetics

To generate clones, we used the *FLP*/*FRT* system driven by an *eyeless* regulatory region, which induces mitotic recombination specifically in the developing eye imaginal disc (Theodosiou and Xu, 1998). This approach produced mosaic eyes containing homozygous mutant and wild-type clones, where they act as controls. The BDSC #43348 stock carries an external photoreceptor regulatory region-driven expression of TdTomato (TdT) on the third chromosome, a fluorescent marker in the third chromosome, allowing distinction between mutant and wild-type photoreceptors, and a transgene in the second chromosome driving expression of GFP in all external photoreceptors. Mutant clones were identified by the absence of the TdT signal. Control clones were generated by crossing BDSC #43348 to BDSC #5619, yielding TdT− and TdT+ wild-type clones (Gambis et al., 2011).

Except for the phototaxis assay, where males and females, separated, less than 5 days old were used, all experiments were performed employing female flies less than 5 days old. Crosses were carried out between BDSC #43348 and either the mutant *kay* alleles or BDSC #5619 as a control. F_1_ flies lacking balancer chromosomes were selected for analysis. These flies were unambiguously identified by the lack of the balancer chromosome dominant marker(s), ensuring these flies carried the chromosome with the *kay* mutation or the control wild-type chromosome and the homologous chromosome with the FRT sequence and the fluorescent marker transgene (Gambis et al., 2011) (see Supplementary Table 2 for detailed crossing schemes).

### 2.3 Optical microscopy image acquisition

Photoreceptor images were acquired on a Zeiss LSM 780 confocal microscope using a LD LCL Plan-Apochromat 25X/0.8 Imm Korr DIC M27 multi-immersion objective, as previously described (Gambis et al., 2011). Live adult flies were anesthetized with CO_2_ and mounted sideways in a drop of 1.5% gelling agarose on a glass slide, with one eye oriented dorsally for imaging. Sample excitation was performed using two laser lines: 488 nm and 561 nm. The 488-nm laser was used with a pinhole setting of 1.69 AU (32 μm) and the 561-nm laser with a pinhole of 1.66 AU (37 μm).

For visualization of *kay* protein-trap expression, third-instar larval eye imaginal discs were dissected in PBS, mounted on glass slides, and imaged using a Zeiss Apotome microscope equipped with a 40 × oil-immersion objective. GFP fluorescence was excited using a 488-nm light source with an appropriate GFP dichroic/filter set.

### 2.4 Scanning electron microscopy

For scanning electron microscopy (SEM), adult flies were fixed as in (Riesgo-Escovar and Hafen, 1997a; Nazario-Yepiz and Riesgo-Escovar, 2017). Briefly, anesthetized flies were fixed in 2% glutaraldehyde and 1% osmium tetroxide in cacodylate buffer and postfixed in 2% osmium tetroxide in the same buffer. Both fixations were performed at 4 °C on ice. They were then dehydrated at room temperature through a graded acetone series, critical-point dried, mounted on stubs with conductive carbon paint, and sputter-coated with gold. Prepared specimens were examined using a JEOL JSM-6060LV SEM microscope under high vacuum at 27 kV, with a spot size of 5.

### 2.5 Image analysis

Image processing and quantitative analyses were performed using Fiji. From confocal datasets, three-dimensional reconstructions were generated from z-stacks using maximum intensity projections. Individual photoreceptor rhabdomeres (the photosensitive organelles in photoreceptor cells) were then identified and marked (segmented) from a single focal plane, and their numbers were quantified using the Cell Counter plugin to be able to identify and perform quantitative analyses.

External eye parameters, such as ommatidial number, bristle morphology, and total eye area, were measured manually in Fiji. To assess the reproducibility of measurements, we measured half of the bristle measurements using the calibration ruler provided in the images by the microscope software, and the other half using fiduciary agents consisting of spherical particles of 3–6 μm in diameter. Individual microspheres were also measured (Supplementary Figure 3F). These microspheres were synthesized from zirconium oxide by Dr. Pedro Salas, CFATA, UNAM (unpublished). We “salted” the critically point-dried and gold-coated specimens with the particles, and afterward did a second, 1-min gold coating of the specimens. They were then observed, and pictures taken where at least one microsphere was in the same frame as the bristle. We used the microsphere diameter to measure the bristles. Measurements with fiduciary agents were not significantly different from the previous series of measurements, so we combined both sets of data (without and with fiduciary agents), as in Supplementary Figure 3A. For analysis of corneal ultrastructure, individual corneal nipples were segmented by supervised delineation, allowing quantification of their shape descriptors and position.

### 2.6 Phototaxis assay

Phototaxis behavior was evaluated using a modified version of the Benzer countercurrent assay (Benzer, 1967). In brief, we used groups of young, same-sex flies (average 35 flies) placed inside a tube. We used both groups of males and females for this test. Once dark-adapted (3 h), we inserted the tube at one end of the countercurrent apparatus and positioned another tube in register with the first one, 30 cm away from the white light source of 150 lux, measured at the decision point of the apparatus. For each trial of the assay, flies were allowed to move to the tube nearer the light source for 1 min. Flies were given two more consecutive opportunities (plus the first one described above) to respond to the light stimulus in the same fashion. The assays were performed at 25 °C, 40–50% relative humidity, starting at the same time always, 12 p.m. At the end of the assay, flies in each tube were counted and scored. Here, we report fly numbers in the last tube, those that responded to the light three times.

### 2.7 Radial distribution function

The spatial distribution of corneal nipples from high-power SEM images was quantified using a radial distribution function (RDF), which describes how point-to-point distances are distributed relative to a random pattern. This metric is widely used in biology to characterize spatial order (He et al., 2023). Nipples´ centroids were used to measure the distance between them using MATLAB to generate density-normalized RDF curves *g* (*r*). To determine whether nipple organization differed from randomness, each dataset was compared with density-matched hard-core simulations, where points are placed randomly but constrained by a minimum separation equal to the average nipple diameter.

Since the nipple distribution is not random, to further quantify spatial organization, we fitted each empirical RDF with a Hard-Core Disk Model, a classical statistical-physics model originally developed to describe systems of non-overlapping particles (Illian et al., 2008). The fitted parameters [minimum spacing (*d*), peak amplitude (*A*), decay length (*λ*), and oscillation period (*P*)] provide interpretable descriptors of nipple packing.

### 2.8 Ommatidial orientation

Ommatidial orientations were assessed by manually measuring the angles formed between R1, R2, and R3 rhabdomeres to the eye equator to record and analyze ommatidial orientation in the eye sections. The developing ommatidia orient with respect to the equator, rotating, with the dorsal and ventral sides having opposed orientations, and so acting as an organizing center. We chose to also refer to it in our measurements as the equator, thus provides a clear guidepost.

To quantify the ommatidial orientation, we calculated the circular mean direction (*μ*) and the length of the mean resultant vector (*R*), which quantifies the concentration of angles around the mean [values range from 0 for uniformly distributed data to 1 for perfect alignment (Berens, 2009)]. Differences in the concentration of angles between wild-type and mutant ommatidia were assessed by comparing resultant vector lengths, while differences in central tendency were assessed by comparing mean directions.

### 2.9 Inverse PCR

To localize the insertion site in *kay^5^*, we performed an inverse PCR protocol using genomic DNA of the *kay^5^* stock and P element 5′ primers, exactly as described in Huang et al. (2009). Briefly, we purified genomic DNA using standard procedures (Sambrook et al., 1989) from the *kay^5^* stock, digested it with frequent cutter restriction enzymes (HinPI, MspI, and Sau3A), ligated the DNA in dilute conditions with T4 ligase, and used the ligations for PCR reactions using the Plac4 and Plac1 primers described in Huang et al. (2009). The resulting amplicon was gel-purified and sequenced in its entirety and mapped onto the genomic sequence (Supplementary Figure 1A).

### 2.10 *In situ* hybridization

In situ hybridization to discs was performed, exactly as described in Dickson et al. (1995), using antisense and sense probes synthesized from a *kay* cDNA (kind gift of Dr. E. Perkins), isolated by Perkins et al. (1990). Briefly, the DIG-RNA probes were synthesized using the Roche *in situ* hybridization kit without digestion. Eye-antennal imaginal discs were dissected from third instar wild type larvae, fixed, and hybridized, as in Dickson et al. (1995), and developed using NBT-BCIP, as recommended by the manufacturer. Discs were then mounted in slides and viewed with a Nikon Eclipse microscope.

### 2.11 Western blotting

Adult flies from the *kay* protein trap and *y.w* controls were probed with an anti-GFP antibody (Santa Cruz Biotechnology #SC-9996 monoclonal antibody). Western blotting was performed as in Riesgo-Escovar et al. (1996). Briefly, we used four adult flies/lane (either *kay* protein trap or *y, w* flies) homogenized in Laemmli 2× buffer, boiled, and loaded onto a 10% acrylamide/bis acrylamide minigel (BioRad). After separation, the gel was transferred to a pure nitrocellulose filter, blocked with BSA, probed with 1:1,000 dilution of the anti-GFP antibody, washed, and then incubated with a 1:5,000 dilution of an anti-mouse secondary antibody coupled to alkaline phosphatase (AP2000, Vector Laboratories) and developed using NBT-BCIP until a clear signal was observed. The filter was then washed with tap water, photographed, and dried.

### 2.12 Code availability

The code used for nipple segmentation, RDF calculation and graphing, and ommatidial orientation quantification is available at https://github.com/Zuniga-Garcia/Kayak_adult_eye_formation_Codes

### 2.13 Statistical analysis

For statistical analysis of ommatidial orientation, we employed the polar statistics MATLAB package and only ommatidia, where all the external photoreceptors were either mutant or wild-type. We analyzed eight eyes per genotype and five ommatidia per eye. For each group of orientations, we calculated the circular mean direction (*μ*) and the length of the mean resultant vector (*R*), which quantifies the concentration of angles around the mean [values range from 0 for uniformly distributed data to 1 for perfect alignment (Berens, 2009)]. Differences in angle concentration between wild-type and *kay* mutant ommatidia were assessed by comparing resultant vector lengths, while differences in central tendency were assessed by comparing mean directions. To test whether the overall distributions of wild-type and *kay* orientations differed, we used Watson’s *U*^2^ two-sample test. This non-parametric test compares the empirical cumulative distribution functions on the circle and evaluates the integrated squared deviation between them, weighted by sample size. Watson’s *U*^2^ does not assume equal dispersion and is sensitive to differences in both location and scale. Statistical significance for all tests was determined using a stratified permutation procedure in which genotype labels were randomly shuffled within each biological replicate (each eye), thereby preserving the nested sampling structure of multiple ommatidial measurements per eye (and 10^4^ permutations per eye). *p*-values were computed with the small-sample correction (*k* + 1)/(*n* + 1) to ensure unbiased estimates.

Experimental radial distribution functions (RDFs) were compared with simulations using the Wilcoxon signed rank test. For comparison of distributions of ommatidial size, we used the Kolmogorov–Smirnov test, a non-parametric test of the equality of continuous probability distributions. All other experiments were analyzed by one- or two-way ANOVA, followed by Dunnett’s *post hoc* test for multiple comparisons. Statistical analyses were performed using GraphPad Prism 9.3.1. Significant thresholds were set at *p* < 0.05, *p* < 0.01, *p* < 0.001, and *p* < 0.0001. All data were plotted using ggplot2 in R Studio, MATLAB, or Prism.

## 3 Results

### 3.1 *kay* is expressed in the developing eye discs

We first examined whether *kay* is normally expressed in the developing eye discs. For this, we performed two tests: we performed *in situ* hybridization to wild-type third instar imaginal eye discs using a probe from the *kay* cDNA isolated and characterized by Perkins et al. (1990), a probe that includes the leucine zipper and basic regions, and so, a probe that hybridizes to all known *kay* transcripts. We found expression in developing ommatidia, behind the morphogenetic furrow (Supplementary Figures 1B,B´). The morphogenetic furrow is an indentation in the surface of the disc proper that travels from posterior to anterior and marks the initiation of differentiation of ommatidia from an otherwise undifferentiated epithelium, starting with the specification of the photoreceptors. We also examined protein expression indirectly by assessing expression of an EGFP-tagged Kay protein in third instar eye discs using a Kay protein trap stock. We found expression in a very similar pattern to the *in-situ* hybridization results in developing ommatidia behind the morphogenetic furrow (Supplementary Figure 1C). We also analyzed a database of single-cell transcriptomes of third instar imaginal eye disc and found *kay* expression in developing photoreceptors (Bozllepogu Raja et al., 2023), in agreement with the *in situ* and protein trap results. Furthermore, consistent with this, we performed a Western blot and found *kay*: EGFP expression in the *kay* protein trap line in adults, and found two distinct bands of apparent 58 and 70 kD (Supplementary Figure 1D). Overall, results show the expression of *kay* in developing eye discs and adults.

### 3.2 *kay* mutations disrupt photoreceptor development

**Figure 1:**
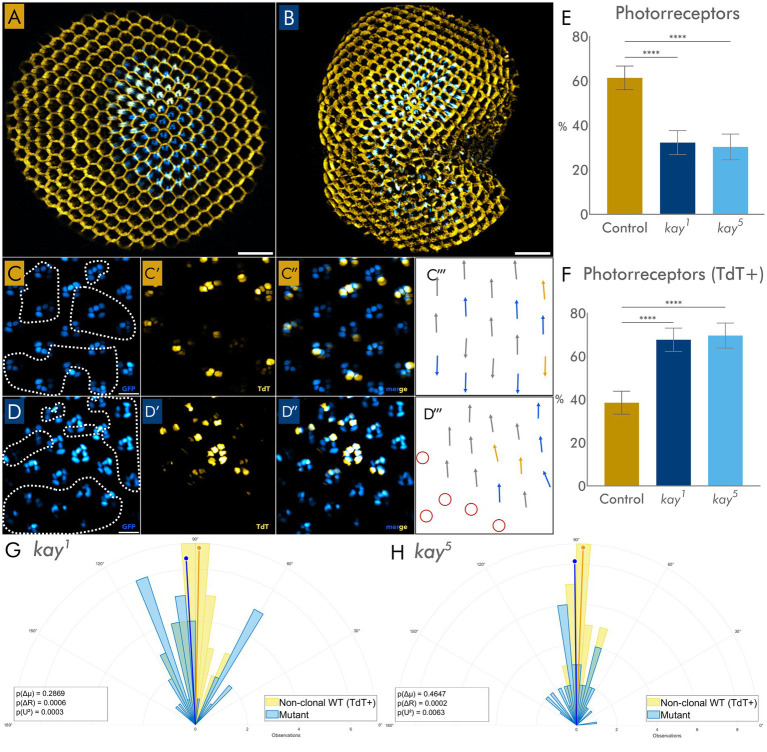
**(A,B)** Confocal reconstructions of eyes with clones. **(A)** Wild-type control clones and **(B)** *kay^1^* mutant clones. **(C)** Focal plane of control ommatidia showing the trapezoidal arrangement of external photoreceptors (PRs). GFP [blue signal in **(C,C″)**] labels all PRs, while TdTomato (yellow) marks wild-type non-clonal rhabdomeres **(C′,C″)**: clonal WT PRs lack TdTomato. **(D)** Equivalent focal plane of a *kay^1^* mutant clone, showing disorganized or missing PRs. GFP [blue signal in **(D,D″)**] labels all PRs, while TdTomato (yellow) marks wild-type rhabdomeres **(D′,D″)**: mutant PRs lack TdTomato. In **C** and **D**, dashed lines outline clonal wild-type (WT) or mutant PR. **(C’’’,D’’’)** Arrows indicate the orientation of individual ommatidia in **C** and **D**. Gray arrows mark ommatidia containing PRs of mixed genotype [mutant or clonal WT (TdT−) and non-clonal WT (TdT+)]. Blue arrows mark ommatidia composed entirely of clonal WT (TdT−) or mutant external PR. Red circles mark ommatidia where it is not possible to measure the orientation due to missing PRs. Only ommatidia with all mutants or all wild-type external PRs were used for the analysis. **(E,F)** Quantification of PRs in eyes with control or mutant clones. *n* = 6 eyes per genotype, with multiple clones per eye. **(E)** A significant difference is shown when comparing the percentage of mutant PR in clones compared to clonal WT, TdT− PR cells in control clones. **(F)** Comparison of non-clonal WT PR **(G,H)**; ommatidial orientation of *kay^1^* **(G)** and *kay^5^* **(H)** mutant ommatidia and wild-type ommatidia. Vectors (colored lines) indicate preferred direction (angle) and tuning strength (length, where *R* is the strength of the preferred direction). The orange line is wild-type and the blue line is mutant. *P*(*U*^2^) indicates the significance obtained by Watson’s *U*^2^ two-sample test. *n* = 11 eyes per genotype, with multiple clones per eye. Statistical significance: **** = *p* < 0.0001. Scale bars: 50 μm **(A,B)**; 10 μm **(C,D)**.

To assess the role of *kay* during eye development, we chose to study the consequences of loss-of-function phenotypes in ocular tissues using mutant alleles of the locus. *kay* loss-of-function alleles are embryonic lethal, so we generated eye clones by means of the *FLP/FRT* technique (Brand and Perrimon, 1993), as implemented for eye clones in Gambis et al. (2011). Mutant (and control) clones were identified by the absence of TdTomato (TdT) fluorescence, allowing us to distinguish them from wild-type photoreceptors (PR). Confocal projections of stacks of optical sections of whole eyes showed the regular, hexagonal arrangement of ommatidia and PR in control eyes and control clones (Figure 1A). The focal planes at the PR level reveal that control external PRs displayed the expected trapezoidal arrangement and wild-type planar cell polarity (Figures 1C’ and C’’). Clones generated with marked wild-type cells had a wild-type phenotype, like wild-type eyes, ensuring that abnormal phenotypes, if any, in eye clones homozygous for *kay* mutations are not due to the clone generating technique.

We first examined mutant eye clones for *kay^2^*, a *kay* hypomorphic mutation. In accordance with published results, *kay^2^* eye clones exhibit near-normal phenotypes (Supplementary Figure 2A); also described in Mitchell et al. (2024). This result is consistent as well with the tenet that the eye clone generating procedure does not alter *per se* the wild-type phenotype of the clonal cells.

We then decided to examine two other *kay* alleles, *kay^1^* (Riesgo-Escovar and Hafen, 1997a) and *kay^5^*, known as strong loss-of-function alleles. In contrast to the *kay^2^* results described above and by Mitchell et al. (2024), homozygosity for *kay^1^* or *kay^5^* eye clones produced irregular ommatidial organization and disrupted external eye morphology (Figures 1B,D; Supplementary Figure 2B). Since *kay^1^* and *kay^2^* were generated in the same mutagenesis (Jurgens et al., 1984), the different results cannot be ascribed to genetic background effects as they share the same genetic background; this is rather consistent with *kay^1^* being a stronger loss-of-function allele of the locus. *kay^1^* and *kay^2^* were backcrossed to the control stock with the FRT82 sequence to recombine the FRT82 onto the chromosomes bearing the *kay* mutations and to have the mutations in a common genetic background since many years had passed since the isolation of the mutations and the present study. Also, *kay^5^* eye clones exhibited very similar eye mutant phenotypes to *kay^1^*, arguing that the phenotype observed in these mutant alleles are due to lack of *kay* function rather than genetic background effects since *kay^1^* and *kay^5^* originated in different mutagenesis, many years apart, and in different labs, and since the *kay^5^* stock was also backcrossed to the same FRT82 chromosome as *kay^1^* and *kay^2^*. We tested this genetic background (the FRT82 stock used) for control eye clones, and these had no discernible mutant phenotypes. For the remainder of the article and for brevity, we will refer collectively to *kay^1^* and *kay^5^* mutant phenotypes as *kay* when phenotypes of both mutants are similar, since both alleles are strong loss-of-function alleles, and to individual mutant alleles when phenotypes differ.

Ommatidia within *kay* mutant clones exhibit disorganized and/or missing PRs (Figures 1D’ and D’’). Quantification of mutant PRs shows a significant reduction in the proportion of mutant PRs compared to controls (Figures 1E,F), and this decrease affected all classes of external PRs equally (Supplementary Figure 2C). This is consistent with mutant PR cells either dying prematurely, leading to ommatidia with missing photoreceptors, or a proportion of mutant PR cells inappropriately specified, not differentiating as PR cells, or both. It is also consistent with a sub-optimal competitiveness of mutant cells in this context.

To evaluate how *kay* mutations may impact planar cell polarity (PCP), we measured ommatidial orientation and compared mutant and wild-type ommatidia in clones (Figures 1C’’’,D’’’). Although the mean mutant ommatidia orientations did not differ from adjacent wild-type ommatidia, the overall orientation distribution of mutant ommatidia was significantly broader than wild-type ommatidia (Figures 1G,H), indicating increased variability in angle orientation and disruption of PCP. Other mutants known to alter PR numbers in ommatidia, such as *phyllopod*, mutant patches of which lack PR1, 6, and 7, show normal PCP (Chang et al., 1995), arguing that changes in PR composition in ommatidia do not necessarily entail PCP changes.

To assess whether this effect extends beyond mutant cells (i.e., whether it was cell autonomous or not), we compared the orientation of wild-type ommatidia adjacent to mutant clones with wild-type ommatidia adjacent to control (clonal wild-type) clones. We found that the adjacent wild-type ommatidia next to mutant ommatidia exhibited a broader orientation distribution compared with clonal or neighboring wild-type ommatidia (Supplementary Figure 2E), consistent with *kay* mutations promoting a non-cell-autonomous disruption of PCP, perturbing both mutant and neighboring wild-type ommatidia.

### 3.3 Eyes with *kay* mutant clones exhibit disrupted external morphology

**Figure 2:**
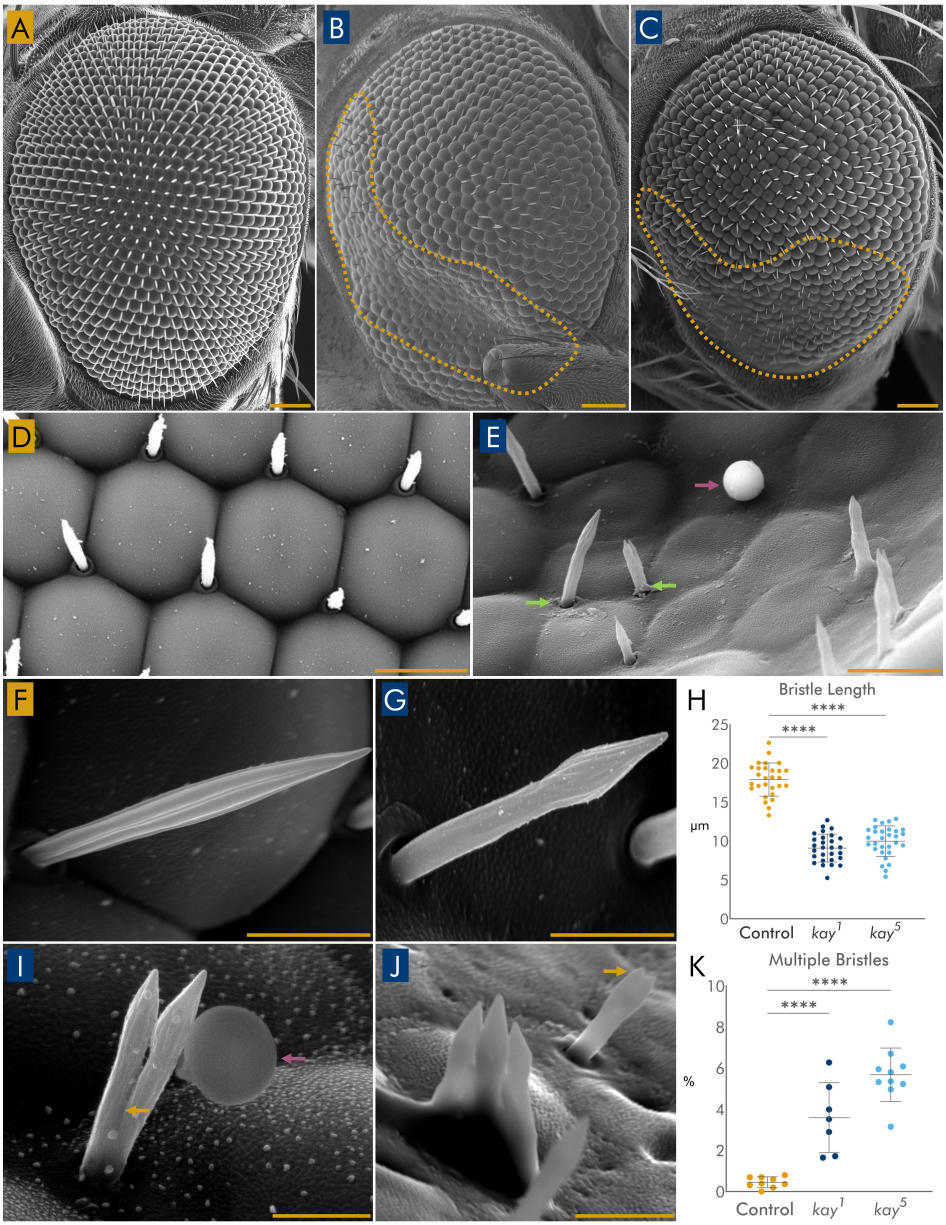
**(A)** SEM of a wild-type eye. **(B,C)** SEM of eyes containing *kay^1^* **(B)** or *kay^5^* **(C)** mutant clones. Yellow dashed lines in each case outline a region with disrupted ommatidial organization. **(D)** High-magnification view of a control eye with wild-type corneal surfaces and regularly spaced bristles. **(E)** Equivalent region in a *kay^1^* mutant clone, at the same magnification, showing a field with several irregular corneal structures and several aberrant bristles. Note a case of aberrant positioning of mutant bristles, marked by green arrows. A fiduciary agent used to measure sizes is marked by a purple arrow. **(F)** Wild-type mechanosensory bristle. **(G)** Representative *kay^1^* mutant bristle displaying the “Paintbrush” phenotype, i.e., enlarged subapical region. **(H)** Quantification of mutants and control bristle length. *n* = 30 bristles from 5 to 6 eyes per genotype. **(I,J)** Examples of a duplicate *kay^5^* bristle with a fiduciary agent (purple arrow) **(I)** and a triplicated *kay^1^* **(J)** bristle with a duplicate bristle in the background. Yellow arrows point to dividing points in mutant bristles in **(I,J)**. **(K)** Quantification of multiple bristles. Triplicated or quadruplicated bristles represent ~3% of the duplicated or more bristles found in eyes with mutant *kay* clones and were not found in control eyes. *n* = 9–11 eyes per genotype. Statistical significance: **** = *p* < 0.0001. Scale bars: 50 μm **(A–C)**; 20 μm **(D,E)**; 5 μm **(F,G,I,J)**.

We next examined in greater detail the external eye structure using SEM. Wild-type eyes displayed the characteristic highly ordered lattice of ommatidia with regularly spaced mechanosensory bristles (Bate and Martinez Arias, 1993) (Figures 2A,D). In contrast, eyes containing *kay* mutant clones showed extensive disruption of ommatidial organization (Figures 2B,C); each dashed outline marks a clonal area within the eye, with regions where the normal hexagonal arrangement was lost. These zones also exhibited a flattening of the corneal structure and abnormally positioned or mis-shaped mechanosensory bristles (Figure 2E). *kay* mutations reduced the total number of ommatidia and bristles, although overall eye size remained unaffected (Supplementary Figures 2G–I).

Mutant bristles were not only irregularly spaced (and some were missing) in the mutant tissue patches, but those present were malformed, with a few even duplicated, triplicated, or more (Figures 2I–K). Although wild-type bristles are long, slender, and taper smoothly to a fine tip (Figure 2F), mutant bristles frequently displayed a distinctive “paintbrush” phenotype, characterized by a bulbous distal region before the fine tip (Figure 2G). Quantification of bristle widths shows that “paintbrush” bristles indeed displayed significantly altered morphology, being thinner at the middle but broadened before the tip (Supplementary Figures 3A–C). in addition, “paintbrush” bristles were significantly shorter than wild-type bristles (Figure 2H). Also, a small but significant percentage of mutant bristles were duplicated or more compared to wild-type bristles (Figure 2K).

### 3.4 Corneal ultrastructure and organization of corneal nipples are disrupted in *kay* mutants

**Figure 3:**
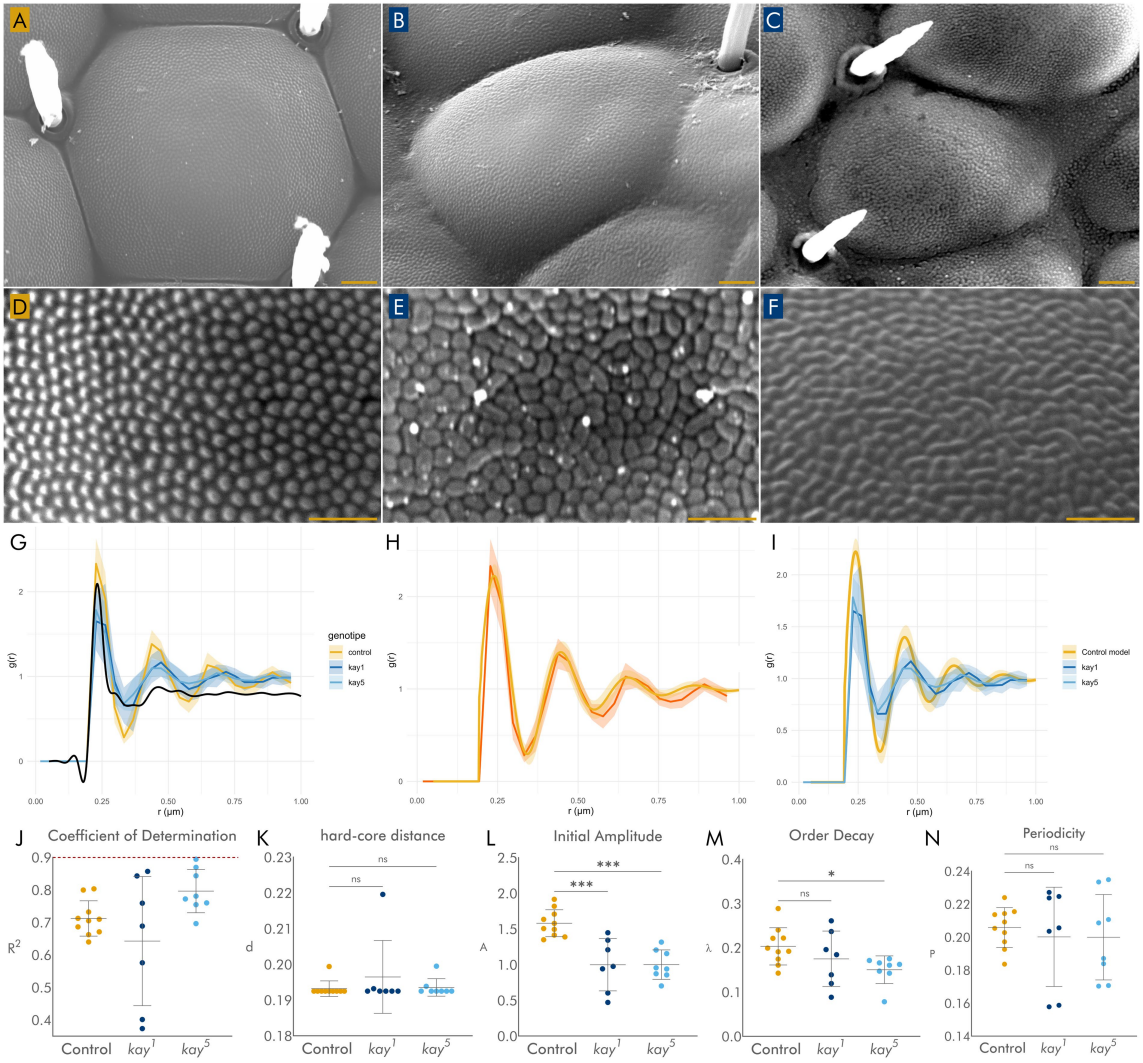
**(A)** Wild-type ommatidium showing corneal surface. (B, C) Mutant *kay^1^* **(B)** and *kay^5^* **(C)** ommatidia with irregular surfaces. **(D)** High-magnification view of wild-type corneal nipples. **(E,F)** High magnification view of mutant *kay^1^* **(E)** and *kay^5^* **(F)** corneal nipples. **(G)** Radial distribution function (RDF) of corneal nipples from control (yellow) eyes and eyes with *kay* mutant clones (blue) compared with simulated random distribution (solid black line). Shaded areas represent standard deviation. *n* = 7–9 ommatidia (each ommatidia from a different eye) per genotype. **(H)** RDF of control nipples (orange) and hard-core disk model (yellow). **(I)** Comparison of control nipples, RDF inferred hard-core disk model with *kay^1^* and *kay^5^* RDFs. **(J)** Coefficient of determination (*R*^2^) values indicating significant deviation of experimental RDFs from random distributions. The red dashed line indicates the lower limit of significance; values under 0.9 are significantly different. **(K–N)** Hard-core disk model parameters of control and mutant RDFs. Significant differences were found with initial amplitude between control and mutant distributions, indicating different separation of corneal nipples. Also, *kay^5^* order decay is significantly different from *kay^1^* and control, indicating overall packaging of corneal nipples is different in *kay^5^*. ns = *p* > 0.05, * = *p* < 0.05, *** = *p* < 0.001, **** = *p* < 0.0001 Scale bars: 2 μm **(A,B)**; 1 μm **(C,D)**.

Using high magnification SEM, we then focused on the organization of the corneal surface. In wild-type eyes, corneas appear hexagonal and regularly convex (Figure 3A), whereas *kay* mutant corneas usually displayed an irregular shape and loss of convexity (Figures 3B,C), and occasionally some corneas even appear fused. Mutant corneal sizes also varied significantly from those of their wild-type counterparts (Supplementary Figure 3D). Quantitative analysis of shape confirmed this disruption: whereas wild-type hexagonal corneas displayed a circularity of ~90%, mutant corneas showed significantly reduced circularity, reflecting loss of their regular hexagonal geometry (Supplementary Figure 3E).

At even higher magnification, wild-type ommatidia exhibited the characteristic nanostructured array of corneal nipples in a uniform pseudo-organized pattern (Gemne, 1966; Blagodatski et al., 2015) (Figure 3D). In contrast, mutant corneas showed corneal nipples with abnormal and disrupted organization (Figures 3E,F). Even though the sizes of *kay* mutant corneal nipples did not vary significantly with respect to wild-type corneal nipples (Supplementary Figure 3G), the mutant *kay^5^* nipple diameters were bigger (Supplementary Figure 3H), and *the kay^1^* circularity was smaller (Supplementary Figure 3F), indicating that the shape of the mutant corneal nipples was different.

To quantify disruptions in corneal nipple organization, we analyzed the spatial arrangement of corneal nipples by calculating their radial distribution function (RDF). In control corneas, the RDF displayed a distribution characterized by a big initial peak, followed by two smaller peaks (Figure 3G, yellow). Compared to an artificially generated random distribution RDF, the RDF observed in control clones is significantly different, showing that the nipple distribution is not random.

In *kay* mutant clones, the initial peak is smaller (compared to the control peak), and the secondary peaks are dampened (again, compared to the control situation) (Figure 3G, blue traces). Comparison with the random distribution generated above shows that these two RDFs have significant differences, also arguing that the RDFs are not random, exhibiting *R*^2^ values lower than 90% (black line) (Figure 3J).

As neither wild-type nor the mutant nipples distribute randomly, we used a hard-core disc model to model the control RDF (Figure 3H). We then compared this model from the control data with the mutant data. We found that they are significantly different, showing that the distribution in the mutants is different from that of the wild-type (Figure 3I). On examining the parameters used to model the RDF, we found that the differences between mutants and control are due mainly to the initial amplitude parameter (Figures 3K–N). The initial amplitude parameter depends on the way individual nipples are packed. In this case, individual mutant nipples are less tightly packed than control ones (Figure 3L). Also, the distribution of first peak values in *kay* mutants was broader, implying greater heterogeneity in nipple spacing relative to the highly uniform wild-type arrangement. Taking all together, this means that besides PR cells, *kay* is also required for corneal and bristle development and differentiation.

### 3.5 Eyes with *kay* mutant clones have significantly altered phototaxis behavior

**Figure 4:**
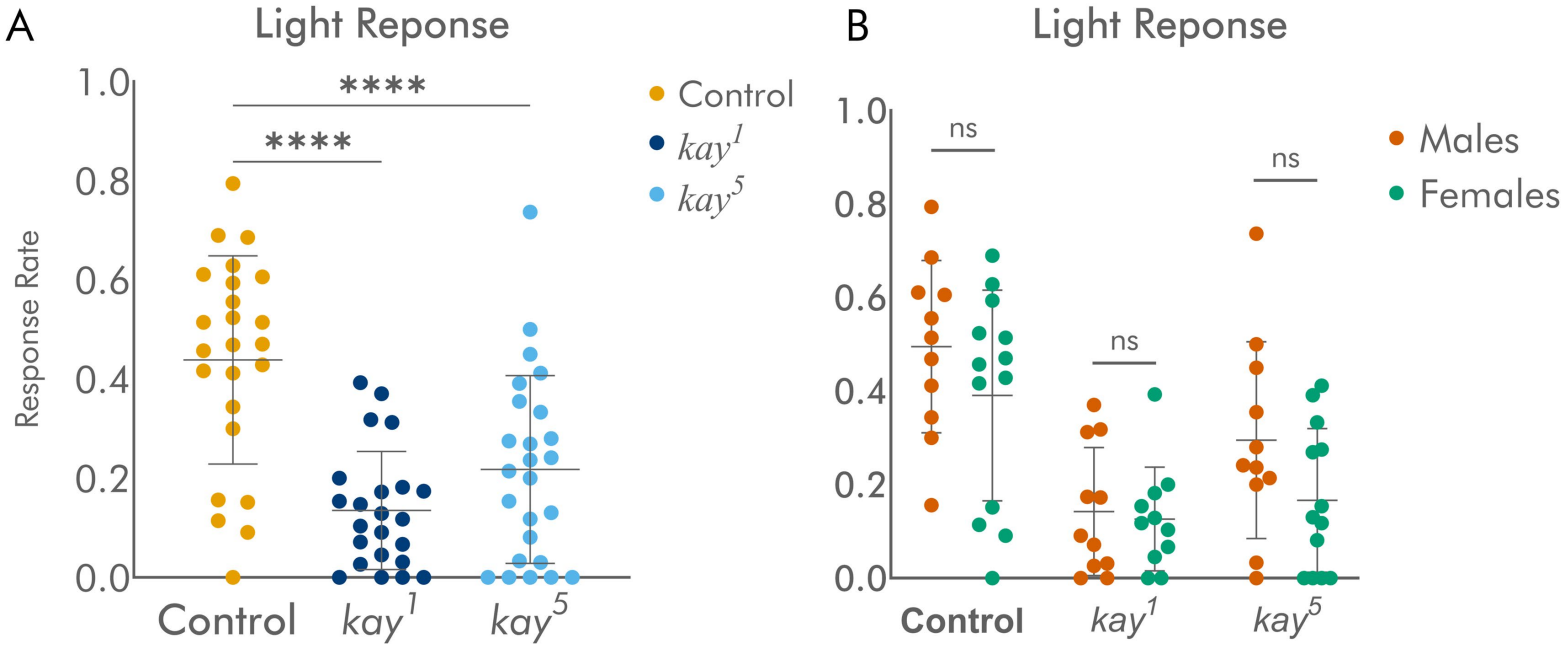
Flies with *kay* mutant eye clones have a significant difference in response to light in a phototaxis assay. **(A)** Quantification of phototaxis behavior in flies with control and *kay* mutant clones. **(B)** Comparison of male and female light responses revealed no significant sex-specific differences. *n* = 24–25 assays per genotype. Statistical significance: ns = *p* > 0.05 **** = *p* < 0.0001.

To determine whether these structural alterations have functional consequences, we tested visual behavior using a simple phototaxis assay. Around 50% of control flies responded three times to the light stimulus and were recovered in the final tube in the countercurrent assay, while *kay* flies had significantly reduced response rates (Figure 4A). The results were similar regardless of whether we used males or females (Figure 4B). These mutant responses for *kay* happen even though only ~30% of the eye is mutant; that means that the eyes harboring mutant *kay* cells also contained wild-type tissue, but the disruptions effected by the mutant tissue were enough to impair normal behavior. Since we used *ey-flp* to generate these mutant clones, we disrupted mainly the eye portion of the eye-antennal imaginal disc. Taken at face value, this is consistent with the need for coherent information from the whole retinal tissue to properly direct behavior. Disruptions of ~30% of the eye territory are enough to derail phototactic behavior significantly. This implies that visual information processing requires coherent input, and that even disruptions in a subset of the visual units are enough to derange decision making and behavioral responses, reminiscent of the frog experiments with a transplanted third eye, where approximately 30% of retinal input is enough to redirect vertebrate optic lobe architecture (Law and Constantine-Paton, 1981).

## 4 Discussion

### 4.1 Fos in fly eye development

We have documented multiple mutant phenotypes in visual tissue deficient in *kayak* function, starting with photoreceptor loss and ommatidial misalignment, bristle and corneal alterations, consistent with *kay* requirements at different stages and cell types during adult eye development. This is consistent with the *fos* function being critical in different guises and in different cellular contexts. These alterations are also reflected in behavioral abnormalities, and since the mutant patches are only generated in the retina, behavioral defects documented here are due to this tissue alone, irrespective of other requirements in other tissues involved in the behavioral output, that is, the brain and muscles.

Since we document here phenotypes only using several loss-of-function alleles, we document requirements free from gain-of-function and artifactual complications, taking advantage of the fact that the fly genome harbors only one *fos* homolog. This enables us to describe the extent of phenotypes and provide a clear picture of *fos* function in this model tissue.

### 4.2 *kayak* locus

The *kayak* locus in *Drosophila* harbors the sole fly *fos* gene homolog (Ozturk-Colak et al., 2024). While this is a reduction in complexity and possible redundancy *vis à vis* other organisms, like vertebrates, it is still a complex locus, coding for five different transcripts, whose theoretical translation products gives rise to five different proteins, sharing a common carboxy-end, where the conserved leucine zipper and basic region are located, but with differing amino terminus, in some cases quite extensive (Hudson and Goldstein, 2008). At least some of these transcripts, in the embryo, are known to be expressed differentially (Dequier et al., 2001). At present, it is unclear whether all *kay* transcripts are expressed in the developing eye, as we used an *in situ* probe that recognizes all of them.

If not all transcripts are expressed or are expressed differentially in different cell types in the developing eye tissue, the ensuing regulation and differential expression may explain the lack of mutant phenotypes in the hypomorphic *kay^2^* allele, as opposed to the *kay^1^* and *kay^5^* results (which behave as strong loss-of-function alleles). It is also conceivable that different cell types (i.e., PR, corneal, and bristle cells) require different forms of Kay. It is not known how the *kay* locus is regulated; what is clear is that it is at least transcriptionally regulated (Hyun et al., 2006). It remains to be seen how *kay* transcripts are regulated during eye development, and how many forms and at what levels they are expressed, notwithstanding the fact that there might be some redundancy between different isoforms.

The foregoing might also mean that expressing ectopically one form of the locus might not fully rescue the mutant phenotype (Riesgo-Escovar and Hafen, 1997a). In addition, gain-of-function experiments can give challenging results to interpret. They might be, at the same time, a gain-of-function for one isoform, but a domineering or dominant negative for others. The resulting phenotype may be a mixture of both gain-of-function and loss-of-function phenotypes (Hyun et al., 2006; Luo et al., 2007; Weber et al., 2008).

### 4.3 *kayak* without *Jra* and the Jun kinase pathway

At present, it is also unclear whether Kay may act alone or in concert with other transcription factor(s) since the leucine zipper is a well-known protein–protein interaction domain. Experiments *in vitro* have shown the feasibility of Kay homodimers (Perkins et al., 1990), but it remains to be seen whether this happens *in vivo*. Since Kay does not require the Jra protein or the jun kinase pathway in the eye for its functions, Kay might act either alone or in conjunction with other transcription factors, different from the classical AP-1 transcription factor complex. In this regard, Kay has been shown to act with Jra and the jun kinase pathway in some instances (Riesgo-Escovar and Hafen, 1997a; Belyaeva et al., 2022; Riese et al., 1997; Szuts and Bienz, 2000; Zeitlinger and Bohmann, 1999), but without them in others (Riesgo-Escovar and Hafen, 1997a; Caputto et al., 2014; Klein et al., 2016; Dequier et al., 2001). It could conceivably act in more than one guise, such as a transcription factor (Perkins et al., 1990), or in lipid synthesis for cell growth in the cytoplasm (Caputto et al., 2014), or as a precursor for piwi RNAs (Klein et al., 2016), and it would be interesting to explore these possibilities.

### 4.4 Planar cell polarity

Planar cell polarity in ommatidial rotation during eye development has been well-documented as a non-cell autonomous phenomenon (Strutt and Strutt, 2021). Both core components of the planar cell polarity network and Notch signaling are instrumental for planar cell polarity in the developing ommatidia in the eye disc (Strutt and Strutt, 2021). In addition, the cuboidal cells (a different tissue from the epithelium proper where the ommatidia are developing), at the border between the epithelium proper and the peripodial membrane of the eye-antennal disc, have also been implicated in ommatidial planar cell polarity (Lim and Choi, 2004; Kumar, 2018). Here, we document planar cell polarity defects not only in the *kay^−^* eye mutant clones but also in neighboring wild-type ommatidia, consistent with the non-cell autonomous behavior of ommatidial rotation and planar cell polarity.

### 4.5 *kayak* in the developing eye

We show here that Kay is required in several instances during eye development, affecting PR, corneal, and bristle cells. It also shows a non-cell-autonomous phenotype with respect to planar polarity in the eye. Taken together, this is consistent with the *kay* expression behind the morphogenetic furrow in developing ommatidia and might point to direct effects in PR cells where *kay* is expressed, but also either to direct or indirect effects in cells differentiating later (cone and bristle cells). It would be of interest to test this by generating the *kay* loss-of-function phenotypes in different cell types, exploring the role of cell autonomy in ommatidial differentiation. Also, it would be of interest to explore whether ectopic cell death occurs during ommatidial development in *kay* clones. In this respect, an ultrastructural study of the developing adult eye would be desirable. Overall, we find here that *kay* is required for the proper development of multiple eye cell types, documenting several *kay* requirements, and thus laying the groundwork for future studies focused on specific phenotypes.

## Data Availability

The code used for nipple segmentation, RDF calculation and graphing, and ommatidial orientation quantification is available at https://github.com/Zuniga-Garcia/Kayak_adult_eye_formation_Codes.

## Supplementary material

Supplementary Figure 1 (A) The *kay^5^* molecular lesion is a transposon insertion in the locus, whereas *kay^1^* is a nonsense mutation. Top: Schematic of the *kay* locus in flies. Colored rectangles indicate exons, while black lines indicate introns. Blue rectangles are non-coding, whereas yellow rectangles are coding. Slanted double lines indicate gaps. 5’ is to the left. The *kay* locus has 7 exons and 5 introns. Triangles mark the point of transposon insertions (*kay^5^* and protein trap). Hexagon marks the nonsense mutation location. The *kay* locus codes for five different transcripts and has four different transcription start sites (Ozturk-Colak et al., 2024). Bottom: Genomic sequence from the first intron of the *kay* locus recovered from the insertion site of the P-element responsible for the *kay^5^* allele. (B) *kay* is expressed in third instar eye discs. *in situ* hybridization of a third instar eye disc showing *kay* mRNA expression behind the morphogenetic furrow (arrows). (B’) sense control of B. Dark patches seen in the border of the eye disc are background staining of NBT-BCIP substrate deposition at the border of the structure. No staining is seen in the central region behind the morphogenetic furrow (arrows). (C) *kay*:GFP protein trap expression in the third instar eye disc. Signal is the GFP fluorescence from the *kay* protein trap, highlighting *kay* presence in developing photoreceptors (arrows). (D) Western blot of control (*y.w*) and *kay*:GFP stocks. Adult flies were homogenized and run in the gel, probed with and anti-GFP antibody. Two bands are seen in the *kay*:GFP, not present in the *y,w* control.

Supplementary Figure 2 (A, B) Focal plane showing *kay^2^* (A) and *kay^5^* (B) mutant ommatidia. GFP (blue in (A, A’’, B, B’’)) labels all PRs, while TdTomato (yellow) marks wildtype rhabdomeres (A’, A’’, B’, B’’): mutant PRs lack TdTomato. Dashed line areas englobe mutant PR. (A’’’, B’’’) Ommatidial orientations of A and B. Arrows indicate the orientation of individual ommatidia. Gray arrows mark ommatidia containing PRs of mixed genotype (mutant (TdT-)) and non-clonal WT (TdT+)). Blue arrows mark ommatidia composed entirely of mutant external PR. Red circles marks ommatidia where it is not possible to measure the orientation due to missing PR. Only ommatidia with all mutants or all wildtype external PRs were used for the analysis. The eye equator is present in both examples, as seen from the orientation of ommatidia. Frequently, mutant *kay* clones span the equator, but often in these clones the loss of photoreceptors is extensive (red circles in B’’’). (C) Quantification of external PRs in eyes with wild-type or mutant clones; no significant differences were observed between the different PRs of the same genotype. Note reduced number of mutant PRs in *kay^1^* and *kay^5^* compared to wildtype PRs (quantitated in figure 1E). *n* = 6 eyes per genotype, but multiple clones per eye. (D-F) Wildtype ommatidia neighboring clonal ommatidia (*n* = 11 eyes with multiple clones per eye per genotype). (D) Orientation of wildtype ommatidia was similar between wild-type clones TdT- (clonal) and TdT+ (non-clonal) photoreceptors in control eyes. (E, F) Orientation distribution of wildtype ommatidia adjacent to *kay^1^* (E) and *kay^5^* (F). Orientation of neighboring wildtype ommatidia to *kay* mutant ommatidia (*kay* adjacent ommatidia) differ significantly from non-neighboring wildtype ommatidia. Vectors (colored lines) indicate preferred direction (angle) and tuning strength (length R). Blue line is wildtype ommatidia neighboring *kay* mutant ommatidia (or purple in D of wildtype ommatidia neighboring wildtype clonal ommatidia), and orange line is non-neighboring wildtype ommatidia. (G) Total number of ommatidia in eyes with control or mutant clones. Eyes with *kay^5^* mutant clones have less ommatidia (*n* = 9-12 eyes per genotype). (H) Number of bristles in eyes with control or mutant clones. Eyes with *kay^5^* mutant clones have fewer bristles. (I) Eye area measurements in eyes with control and mutant clones. No significant differences were noted. (J) Rate of bristles per ommatidia shows that *kay^5^* has lower rate than control and *kay^1^*. (C, G-J) Each data point represents an animal. (D-F) Data from 10 animals was quantitated. Statistical significance: ** = *p* < 0.01, **** = *p* < 0.0001. Scale bars: 50 μm (A).

Supplementary Figure 3 (A) Quantification of bristle widths at the base, middle and subapical regions. Significant widths differences between control and mutants occur at all measurements. Lines linking yellow squares, green circles and pink triangles indicate measurements form the same bristle for all genotypes. *n* = 54-56 bristles per genotype, from 10 different eyes per genotype. (B, C) Representative wild-type (B) and mutant (C) bristles with lines indicating measurement positions. (D) Individual corneal size. Each point represents a cornea. # indicates statistical difference (*p* = 0.0074) in the distribution of *kay^1^* ommatidial size compared with control assessed by Kolmogorov-Smirnov test. *N* = 48-52 corneas for 8 different eyes per genotype. (E) Quantification of corneal circularity. (F-H) Shape descriptors of corneal nipples: (F) circularity, (G) area, and (H) diameter. (A, D, E) Each data point represents a bristle or a cornea. (F-H) Each data point represents the mean of ~400 nipples per animal, *n* = 7-9 different animals per genotype. (I) Sizes of fiduciary agents used; there were no statistical differences between fiduciary agents measured in different phenotypes. Statistical significance: ns = *p* > 0.05, * = *p* < 0.05, and **** = *p* < 0.0001.

Supplementary Table 1 Genotypes of fly stocks used. The complete genotypes of all the stocks used in the paper are listed according to Flybase nomenclature. N/A= not applicable. TM3= Third Multiple 3, a balancer chromosome. TM6B= Third Multiple 6B, a balancer chromosome. For genetic nomenclature and usage, please refer to (Ozturk-Colak et al., 2024).

Supplementary Table 2 Genetic crosses. Listed are the genetic crosses performed to obtain eye clones described in the paper. For nomenclature, see (Ozturk-Colak et al., 2024). Male and female parental genotypes are listed, as well as female F1 offspring used. “Figure” lists where the different female F1 are shown. Supplementary figures are marked starting with a “S”.
