# Supplementary figures and images for: The fos homolog kayak is required for adult eye formation and function in Drosophila
Source: Front Neurosci. 2026 Jan 14;19:1703753. doi: 10.3389/fnins.2025.1703753 (PMC12851592; doi:10.3389/fnins.2025.1703753)

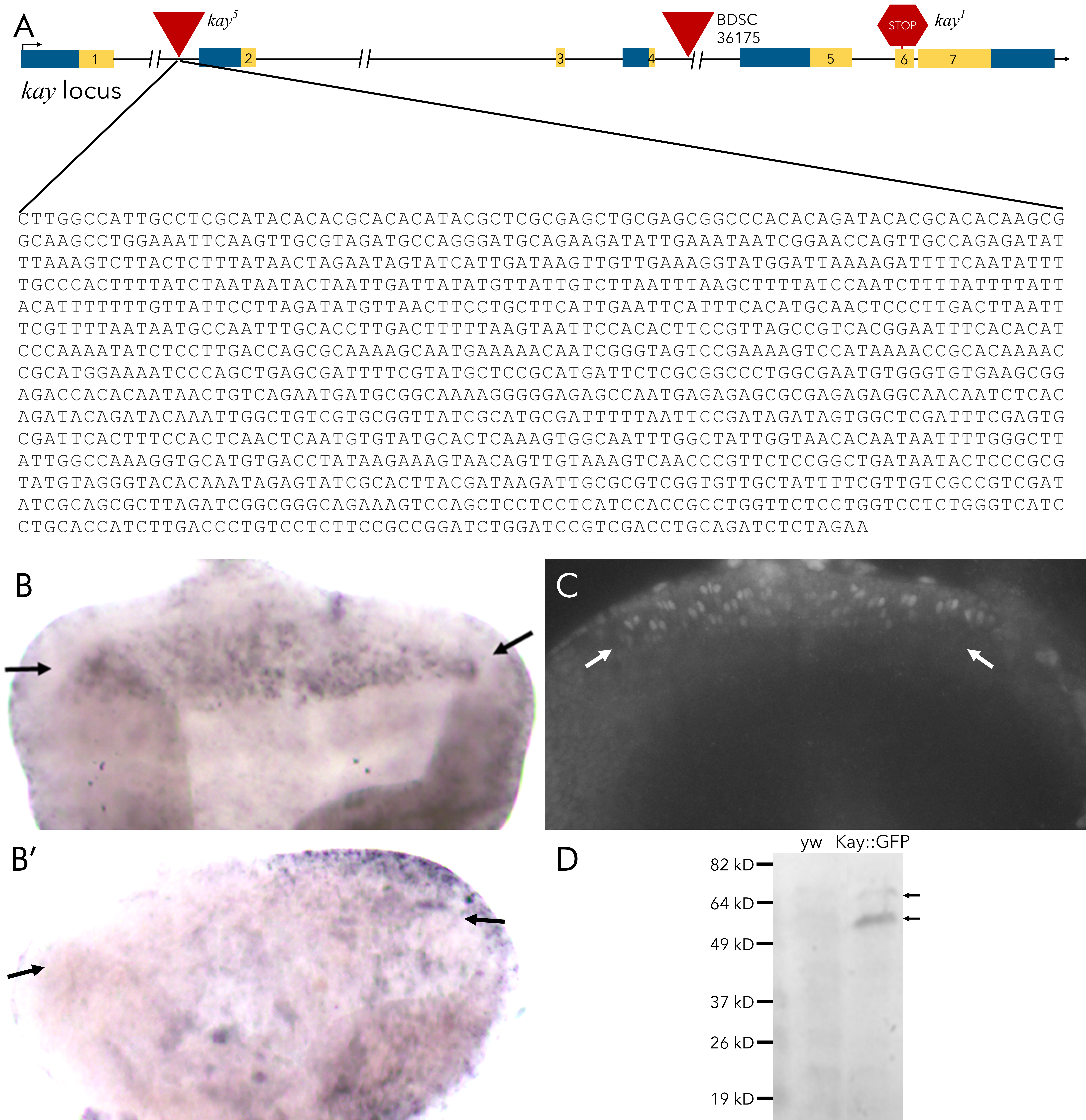

Supplement: Supplementary Figure 1 — (A) The kay5 molecular lesion is a transposon insertion in the locus, whereas kay1 is a nonsense mutation. Top: Schematic of the kay locus in flies. Colored rectangles indicate exons, while black lines indicate introns. Blue rectangles are non-coding, whereas yellow rectangles are coding. Slanted double lines indicate gaps. 5’ is to the left. The kay locus has 7 exons and 5 introns. Triangles mark the point of transposon insertions (kay5 and protein trap). Hexagon marks the nonsense mutation location. The kay locus codes for five different transcripts and has four different transcription start sites (Ozturk-Colak et al., 2024). Bottom: Genomic sequence from the first intron of the kay locus recovered from the insertion site of the P-element responsible for the kay5 allele. (B) kay is expressed in third instar eye discs. in situ hybridization of a third instar eye disc showing kay mRNA expression behind the morphogenetic furrow (arrows). (B’) sense control of B. Dark patches seen in the border of the eye disc are background staining of NBT-BCIP substrate deposition at the border of the structure. No staining is seen in the central region behind the morphogenetic furrow (arrows). (C) kay:GFP protein trap expression in the third instar eye disc. Signal is the GFP fluorescence from the kay protein trap, highlighting kay presence in developing photoreceptors (arrows). (D) Western blot of control (y.w) and kay:GFP stocks. Adult flies were homogenized and run in the gel, probed with and anti-GFP antibody. Two bands are seen in the kay:GFP, not present in the y,w control. [file Image_1.TIF]

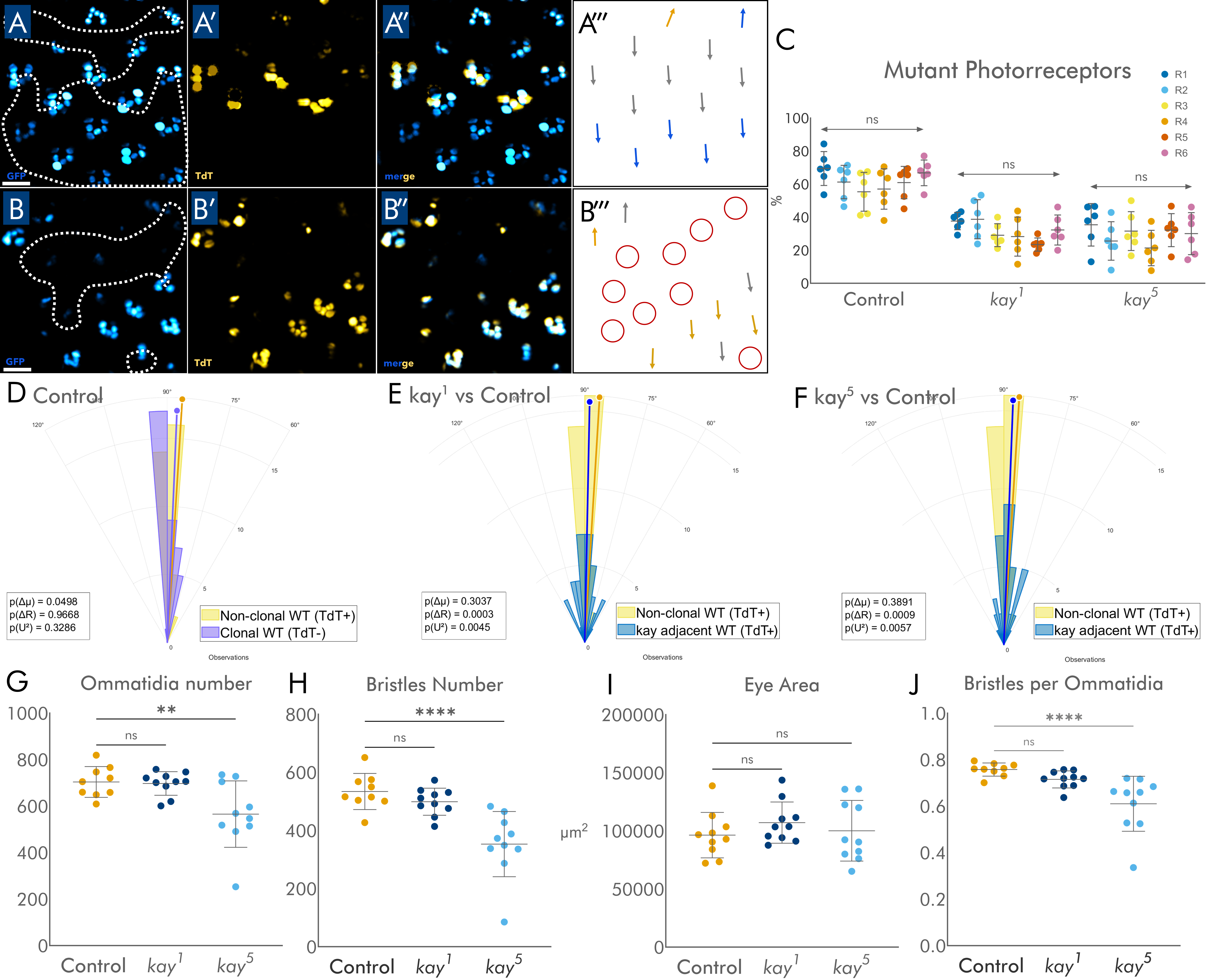

Supplement: Supplementary Figure 2 — (A, B) Focal plane showing kay2 (A) and kay5 (B) mutant ommatidia. GFP (blue in (A, A’’, B, B’’)) labels all PRs, while TdTomato (yellow) marks wildtype rhabdomeres (A’, A’’, B’, B’’): mutant PRs lack TdTomato. Dashed line areas englobe mutant PR. (A’’’, B’’’) Ommatidial orientations of A and B. Arrows indicate the orientation of individual ommatidia. Gray arrows mark ommatidia containing PRs of mixed genotype (mutant (TdT-)) and non-clonal WT (TdT+)). Blue arrows mark ommatidia composed entirely of mutant external PR. Red circles marks ommatidia where it is not possible to measure the orientation due to missing PR. Only ommatidia with all mutants or all wildtype external PRs were used for the analysis. The eye equator is present in both examples, as seen from the orientation of ommatidia. Frequently, mutant kay clones span the equator, but often in these clones the loss of photoreceptors is extensive (red circles in B’’’). (C) Quantification of external PRs in eyes with wild-type or mutant clones; no significant differences were observed between the different PRs of the same genotype. Note reduced number of mutant PRs in kay1 and kay5 compared to wildtype PRs (quantitated in figure 1E). n = 6 eyes per genotype, but multiple clones per eye. (D-F) Wildtype ommatidia neighboring clonal ommatidia (n = 11 eyes with multiple clones per eye per genotype). (D) Orientation of wildtype ommatidia was similar between wild-type clones TdT- (clonal) and TdT+ (non-clonal) photoreceptors in control eyes. (E, F) Orientation distribution of wildtype ommatidia adjacent to kay1 (E) and kay5 (F). Orientation of neighboring wildtype ommatidia to kay mutant ommatidia (kay adjacent ommatidia) differ significantly from non-neighboring wildtype ommatidia. Vectors (colored lines) indicate preferred direction (angle) and tuning strength (length R). Blue line is wildtype ommatidia neighboring kay mutant ommatidia (or purple in D of wildtype ommatidia neighboring wildtype clonal ommatidia), and [file Image_2.TIF]

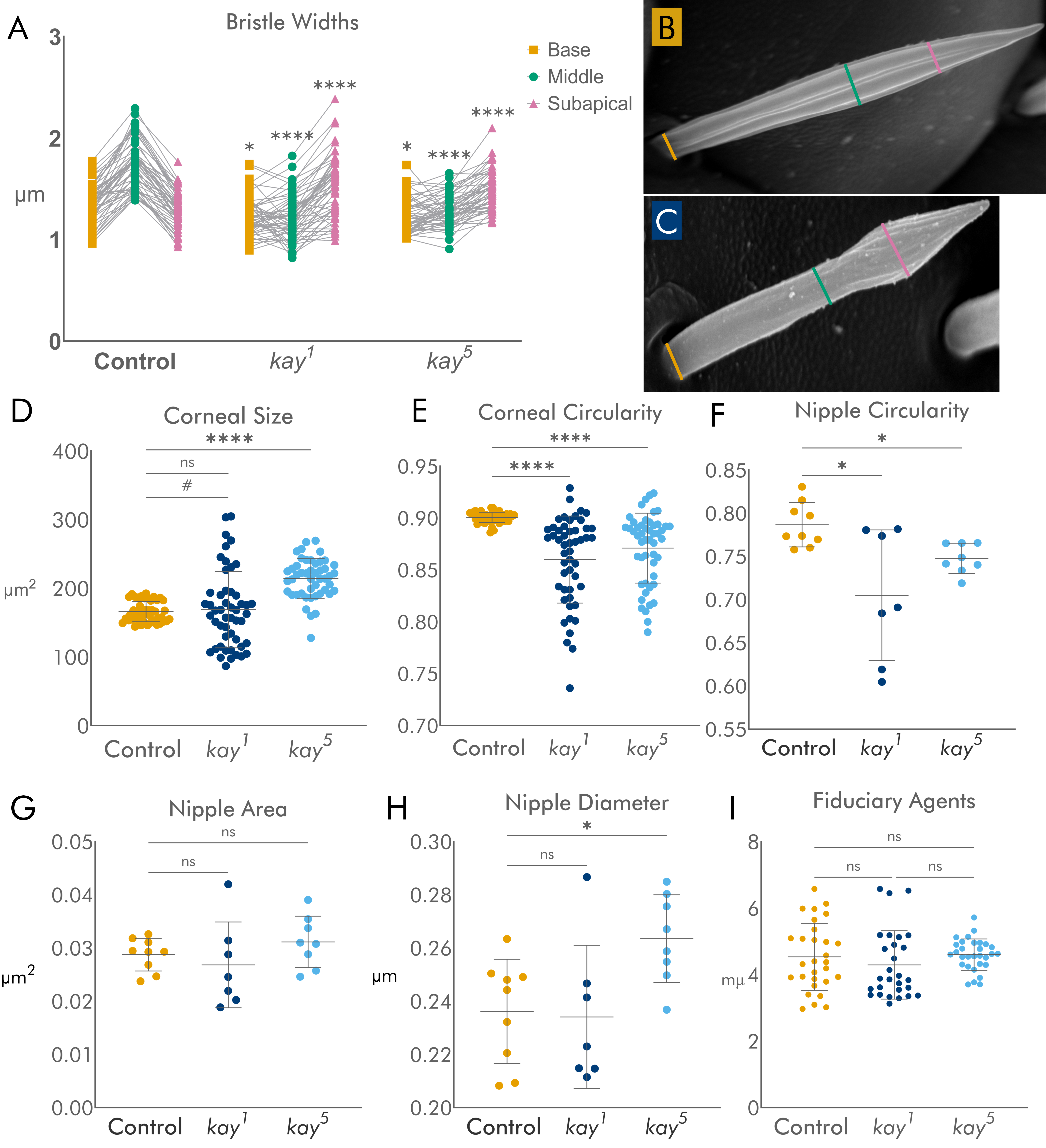

Supplement: Supplementary Figure 3 — (A) Quantification of bristle widths at the base, middle and subapical regions. Significant widths differences between control and mutants occur at all measurements. Lines linking yellow squares, green circles and pink triangles indicate measurements form the same bristle for all genotypes. n = 54-56 bristles per genotype, from 10 different eyes per genotype. (B, C) Representative wild-type (B) and mutant (C) bristles with lines indicating measurement positions. (D) Individual corneal size. Each point represents a cornea. # indicates statistical difference (p = 0.0074) in the distribution of kay1 ommatidial size compared with control assessed by Kolmogorov-Smirnov test. N = 48-52 corneas for 8 different eyes per genotype. (E) Quantification of corneal circularity. (F-H) Shape descriptors of corneal nipples: (F) circularity, (G) area, and (H) diameter. (A, D, E) Each data point represents a bristle or a cornea. (F-H) Each data point represents the mean of ~400 nipples per animal, n = 7-9 different animals per genotype. (I) Sizes of fiduciary agents used; there were no statistical differences between fiduciary agents measured in different phenotypes. Statistical significance: ns = p > 0.05, * = p < 0.05, and **** = p < 0.0001. [file Image_3.TIF]
