# Supplementary material for: The fos homolog kayak is required for adult eye formation and function in Drosophila
Source: Front Neurosci. 2026 Jan 14;19:1703753. doi: 10.3389/fnins.2025.1703753 (PMC12851592; doi:10.3389/fnins.2025.1703753)
Supplement: Supplementary Table 1 — Genotypes of fly stocks used. The complete genotypes of all the stocks used in the paper are listed according to Flybase nomenclature. N/A= not applicable. TM3= Third Multiple 3, a balancer chromosome. TM6B= Third Multiple 6B, a balancer chromosome. For genetic nomenclature and usage, please refer to (Ozturk-Colak et al., 2024). [file Table_1.docx]

| Parental genotypes | | F_1_ Selected Genotype (female) | Figure(s) |
| --- | --- | --- | --- |
| Male | Female |  |  |
| *y^d2^ w^1118^* P{ey-FLP.N}2 P{5xglBS-lacZ.38-1}TPN1; P{neoFRT}82B | P{rh1-GAL4}1, P{ey-FLP.N}2; P{UAS-GFP-ninaC}2; P{neoFRT}82B P{ninaE-tdTomato-ninaC}3R/TM6B, *Tb^1^* | P{rh1-GAL4}1, P{ey-FLP.N}2/ *y^d2^ w^1118^* P{ey-FLP.N}2 P{5xglBS-lacZ.38-1}TPN1; P{UAS-GFP-ninaC}2/+; P{neoFRT}82B P{ninaE-tdTomato-ninaC}3R/ P{neoFRT}82B | 1A, C, E, F;  2A, D, F, H, K;  3A, D, G-N;  4A, B;  S2C, D, G-J;  S3A, B, D-H. |
| *y^*^ w^*^*; p{neoFRT}82B *kay^1^*/TM3 *Sb^1^ Ser^1^* | P{rh1-GAL4}1, P{ey-FLP.N}2; P{UAS-GFP-ninaC}2; P{neoFRT}82B P{ninaE-tdTomato-ninaC}3R/TM6B, *Tb^1^* | P{rh1-GAL4}1, P{ey-FLP.N}2/ *y^*^ w^*^*; P{UAS-GFP-ninaC}2/+; p{neoFRT}82B *kay^1^*/ P{neoFRT}82B P{ninaE-tdTomato-ninaC}3R | 1B, D, E, F, H;  2B, G H, J, K;  3B, E, G, I-N-G;  4A, B;  S2C, E, G-J;  S3A, C, D-H.. |
| *y^*^ w^*^*; p{neoFRT}82B, *kay^5^* /TM3 *Sb^1^ Ser^1^* | P{rh1-GAL4}1, P{ey-FLP.N}2; P{UAS-GFP-ninaC}2; P{neoFRT}82B P{ninaE-tdTomato-ninaC}3R/TM6B, *Tb^1^* | P{rh1-GAL4}1, P{ey-FLP.N}2/ *y^*^ w^*^*; P{UAS-GFP-ninaC}2/+; p{neoFRT}82B,  *kay^5^*/ P{neoFRT}82B P{ninaE-tdTomato-ninaC}3R | 1E, F, I;  2C, I, H, K;  3C, F, G, I-N;  S2B, C, F, G-J;  S3A, D-H. |
| P{neoFRT}82B *kay^2^* *ca^1^*/TM6B, *Tb^1^ ca^1^* | {rh1-GAL4}1, P{ey-FLP.N}2; P{UAS-GFP-ninaC}2; P{neoFRT}82B P{ninaE-tdTomato-ninaC}3R/TM6B, Tb^1^ | P{rh1-GAL4}1, P{ey-FLP.N}2/+; P{UAS-GFP-ninaC}2/+; p{neoFRT}82 kay^2^ ca^1^/ P{neoFRT}82B P{ninaE-tdTomato-ninaC}3R | S2A |

**Supplementary Table 2. Genetic crosses.**
